# A novel mouse model for cardiovascular-kidney-metabolic syndrome: Bridging metabolic, renal and cardiac dysfunction

**DOI:** 10.1016/j.molmet.2026.102368

**Published:** 2026-04-20

**Authors:** Arianne van Koppen, José A. Inia, Romer A. Gonzalez-Villalobos, Anke M. Smits, Andrea R. Nawrocki, Simon A. Hinke, Joline Attema, Christa de Ruiter, Tri Q. Nguyen, Amelie Dendooven, Ingeborg Bajema, Harry van Goor, Toon A.B. van Veen, Willem B. van Ham, Felix Eichinger, Søren H. Elsborg, Henricus A.M. Mutsaers, Elsbet J. Pieterman, Aswin L. Menke, Matthew D. Breyer, Reinout Stoop

**Affiliations:** 1 Department of Metabolic Health Research, The Netherlands Organization for Applied Scientific Research (TNO), Leiden, the Netherlands; 2 Johnson & Johnson Innovative Medicine, Cambridge, MA, United States of America; 3 Department of Cell and Chemical Biology, Leiden University Medical Center, Leiden, the Netherlands; 4 Department of Pathology, University Medical Center Utrecht (UMCU), Utrecht, the Netherlands; 5 Department of Pathology, University Medical Center Antwerp, Antwerp, Belgium; 6 Department of Pathology and Medical Biology, University Medical Center Groningen, Groningen, the Netherlands; 7 Department of Medical Physiology, UMCU, the Netherlands; 8 Department of Internal Medicine, University of Michigan, Ann Arbor, MI, United States of America; 9 Department of Clinical Medicine, Aarhus University, Aarhus, Denmark

**Keywords:** Cardiovascular-kidney-metabolic syndrome, Progressive diabetic kidney disease, GFR decline, Glomerulosclerosis, HFpEF

## Abstract

**Background:**

CKM syndrome involves obesity, type 2 diabetes (T2D), chronic kidney disease (CKD) and cardiovascular disease (CVD). However, most preclinical models fail to reproduce the progressive renal and cardiac dysfunction characteristic of advanced CKM syndrome, limiting their ability to accurately reflect human disease.

**Methods:**

Male uninephrectomized (UNx) KK-Ay mice received a high-fat diet (HFD) with or without the vasoconstrictor L-NNA for 13–16 weeks.

**Results:**

UNx + HFD + L-NNA resulted in obesity, hyperglycemia and progressive kidney failure, indicated by a rapid increase in albuminuria and transient hyperfiltration followed by progressive glomerular filtration rate (GFR) decline over three months. Histopathological analysis revealed severe glomerular damage, fibrosis, inflammation and basement membrane thickening, most pronounced in UNx + HFD + L-NNA mice. Renal transcriptomics analysis revealed robust activation of inflammatory and fibrotic pathways, again most pronounced in UNx + HFD + L-NNA mice.

In the heart, UNx + HFD + L-NNA resulted in increased ejection fraction and fractional shortening, reduced end-systolic volume and increased left ventricular posterior wall thickness. Alongside pronounced right ventricular fibrosis, this phenotype points toward a phenotype of heart failure with preserved ejection fraction (HFpEF).

**Conclusions:**

The UNx + HFD + L-NNA KK-Ay model reproduces key metabolic, renal and cardiac components of CKM syndrome. While obesity and hyperglycemia contribute substantially to disease burden, L-NNA-induced hypertension further exacerbates both renal decline and cardiac remodeling. Therefore, this model enables mechanistic investigation and evaluation of therapeutic strategies for CKM syndrome.

## Highlights

• This study addressed the lack of preclinical models reflecting metabolic, renal and cardiac dysfunction in CKM syndrome.
• We tested a multifactorial mouse model combining uninephrectomy, high fat diet and vasoconstriction to mimic CKM syndrome progression.
• In KK-Ay mice, this induced progressive kidney disease and HFpEF-like cardiac changes with inflammation and fibrosis.
• This model enables both mechanistic investigation and therapeutic exploration of CKM syndrome.

## Graphical abstract

**A multifactorial mouse model of cardiovascular-kidney-metabolic (CKM) syndrome.** The model combines the KK-Ay genotype with uninephrectomy (UNx), high fat diet (HFD) and vasoconstriction (by L-NNA) to induce integrated dysfunction across three organ systems. At the cardiovascular level, a phenotype resembling heart failure with preserved ejection fraction (HFpEF) is observed, characterized by maintained cardiac output, increased fractional shortening, elevated ejection fraction and cardiac fibrosis. At the kidney level, there is a progressive decline in glomerular filtration rate (GFR) accompanied by marked albuminuria, glomerular damage, inflammation and fibrosis. Metabolically, the model develops obesity, hyperglycemia and mild hyperlipidemia develop in this mouse model. This multifactorial approach enables mechanistic studies of diabetic kidney disease progression within the CKM syndrome context.

**Figure:**
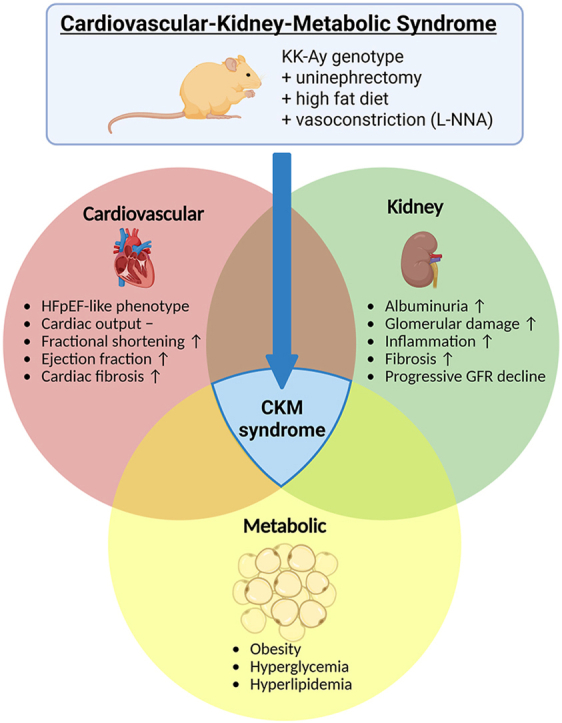

## 1 Introduction

In 2023, the American Heart Association recognized cardiovascular-kidney-metabolic (CKM) syndrome as a distinct disorder characterized by the coexistence of obesity, type 2 diabetes (T2D), chronic kidney disease (CKD) and cardiovascular disease (CVD) [1]. Over a quarter of U.S. adults suffer from at least one CKM component and ∼8% experience multimorbidity [2]. Nevertheless, these conditions are often treated separately, resulting in fragmented care, missed opportunities for early intervention, and increased healthcare costs [1].

Progress in CKM therapeutics requires preclinical models that reflect the interconnected metabolic, renal and cardiovascular abnormalities seen in patients. Although diabetic kidney disease (DKD) is a central feature of CKM syndrome and model evaluation has been guided by criteria established by the Animal Models of Diabetic Complications Consortium (AMDCC) [3,4], most existing mouse models fail to reproduce the full progression to renal and cardiac dysfunction [5]. A major challenge remains the development of a multifactorial model in which CKM risk factors collectively drive progressive renal and cardiac impairment.

To address this gap, we established a CKM syndrome mouse model integrating metabolic, renal and cardiovascular features. KK-Ay mice, which naturally develop obesity and T2D [6,7], were further challenged by uninephrectomy, high fat diet (HFD) feeding and L-NNA-induced hypertension. This combination resulted in a phenotype closely resembling human CKM syndrome, including T2D, progressive renal insufficiency and a cardiovascular phenotype suggesting heart failure with preserved ejection fraction (HFpEF). This model provides a robust platform for testing CKM syndrome-targeted interventions and for investigating mechanisms that link metabolic, renal, and cardiac dysfunction.

## 2 Methods

### 2.1 Animals and welfare

Experimental procedures were approved by The Netherlands Central Authority for Scientific Procedures on Animals (CCD; project license AVD5010020172064) and independent Animal Welfare Body of TNO (IvD TNO; TNO-464, -490, -516). Male KK.Cg-Ay/J (KK-Ay, stock No: 002468) mice (4–7 weeks old) were obtained from The Jackson Laboratory (Bar Harbor, ME, USA) and housed at Innoser Laboratories (Leiden, the Netherlands) under controlled temperature, 12h light–dark cycle, 50–60% humidity and ad libitum access to food and heat-sterilized water. Male mice were chosen because of their greater susceptibility to hyperglycemia, obesity and renal failure (unpublished in-house data and [[8], [9], [10]]).

KK-Ay mice require several husbandry adaptations to ensure optimal welfare. Because of impaired wound healing and diabetic ulcer development, worsened by increased fighting behavior, mice were housed individually in half-heated cages (Tecnilab-Bmi BV, Someren, the Netherlands) supplemented with additional nesting material, wooden chewing sticks and cardboard hut. Wound development was further minimized by refined tube-handling, reducing handling procedures and optimizing diabetic wound care.

### 2.2 Uninephrectomy

Analgesia was started one day before surgery using Carprofen (1175158 Rimadyl Cattle; 5 μL/g body weight, 0.067 mg/mL in drinking water; Zoetis, Capelle aan den IJssel, the Netherlands), continued until day 7 post-surgery, and was then reduced to 2.5 μL/g body weight for four additional days. Ten minutes before surgery, mice received a subcutaneous injection of fentanyl (0.06 mg/kg; Hameln Pharma GmbH, Hamelin, Germany) and midazolam (Dormicum; 0.2 mg/kg, Aurobindo Pharma BV, Baarn, the Netherlands). Anesthesia was induced by isoflurane (2% isoflurane, 0.4 L/min O_2_ and 0.8 L/min air). Eyes were covered with eye lubricant (AA-Retinol 15 Oogzalf; Medpets, Roosendaal, the Netherlands) and skin was shaved and disinfected. A 1.5 cm dorsal incision was made 1 cm below the ribs. The peritoneum was anesthetized using 2% lidocaine (Xylocaine 10% Spray solution (PharmaMarket, ASPEN Pharma Trading Limited, Dublin, Ireland) and 0.5% bupivacaine solution (Marcaine 5 mg/mL; Covetrus, Cuijk, the Netherlands). An incision was made using a dorsal approach. The left kidney was located, adrenal gland separated, and the kidney was ligated with non-absorbable ligature and removed. The peritoneum was closed with absorbable ligatures, the skin cleaned with saline, sutured, and stapled with 9 mm autoclips. Postoperatively, mice received 1 mL warm saline (i.p.) and 2.5 mg/kg Carprofen (s.c.). Animals recovered for 3–4 weeks before starting dietary interventions (week 0).

### 2.3 Experimental design

Three animal studies were conducted (Supplemental Figure S1). Study 1 was a metabolic and renal characterization study. Four weeks post-uninephrectomy (UNx) (week 0), mice were matched into equal groups based on body weight, age, blood glucose and glomerular filtration rate (GFR). One group continued on chow (V1534, Ssniff, Woerden, the Netherlands), a second group was switched to high fat diet (HFD; D12451, Research Diets, New Brunswick, NJ, USA) and a third group was switched to HFD and provided with N^G^-Nitro-l-arginine hydrochloride in the drinking water (L-NNA; 50 mg/L; N5501, Sigma–Aldrich, St. Louis, MO, USA) for 16 weeks. Study 2 was a renal function validation study, where mice received UNx + HFD + L-NNA for 20 weeks. Study 3 focused on cardiac function and included a chow control group, a UNx + HFD group and UNx + HFD + L-NNA group. Across studies, body weight and food/water intake were determined weekly. Plasma, urine and GFR were collected at specific timepoints (Supplemental Figure S1). Mice were sacrificed under isoflurane anesthesia and perfused with PBS/heparin solution (5 mL/min, 90 mmHg, 5 IU/mL) under deep isoflurane anesthesia using an Autoperfuser™ (OS2100; Osenses, Adelaide, South Australia, Australia).

### 2.4 Blood and urine analyses

Blood samples were collected from the tail vein of 4-hour-fasted mice into EDTA-coated tubes. Blood glucose was determined during blood collection using a hand-held glucometer (Freestyle Freedom Light; Abbott Laboratories, Lake Bluff, IL, USA). Plasma insulin and cholesterol were determined as previously described [11]. Urine was collected in the morning by placing mice on hydrophobic LabSand (Braintree scientific, INC., Braintree, MA, USA) for 3 h. Urinary volume was determined and diuresis calculated as volume per 3 h. Urinary cystatin C and nephrin were measured by ELISA (#1043 and #1037, respectively; Ethos Biosciences, Logan Township, NJ, USA). Urinary albumin was measured by ELISA (#80630; Crystal Chem, Elk Grove Village, IL, USA) and urinary creatinine by using the Creatinine Companion Assay (#1012, Ethos Biosciences), followed by calculation of the urinary albumin/creatinine ratio.

### 2.5 Glomerular filtration rate

One day before GFR measurement, mice were anaesthetized (2% isoflurane) and the dorsal rump was shaved and depilated using hair-removal cream (Veet for sensitive skin; Reckitt Benckiser Group PLC, Slough, UK). On the day of measurement, mice were mildly sedated with isoflurane and a transdermal mini-device (MediBeacon GmbH, Mannheim, Germany) was attached to the dorsal rump using an adhesive patch (MediBeacon GmbH). Mice received a retro-orbital injection containing FITC sinistrin (25 mg/mL in saline; 2.8 μL/g body weight; MediBeacon GmbH), and eyes were moistened with Opticorn AD eye ointment (Ecuphar, Oudenaarde, Belgium). The device measured transdermal fluorescence every second for 2 h. After sensor removal, skin was treated with PUUR® wound gel. GFR curves were generated using MBLab (BStudio version 2; MediBeacon).

### 2.6 Cardiac function

Cardiac function was assessed in five mice per group selected to represent the group averages for body weight, plasma lipids, blood glucose and GFR. Animals were anesthetized (isoflurane; 2.5–3% induction; 1.5–2.5% maintenance) and kept on a heating pad. Ultrasound measurements were performed with a VEVO 3100 machine and MX550D probe (Fujifilm, Visualsonics). Mice were depilated, placed in supine position and monitored for heart rate, respiration, and temperature (maintained at 37 °C). Only short axis measurements were analyzed, as papillary muscles interfered with long-axis quantification, although long-axis images showed similar patterns. Short-axis acquisitions included B-mode, M-Mode, and EKV at the level of papillary muscles, and a 4D rendering was obtained for overall assessment. Images were analyzed using VevoLab software (Fujifilm, Visualsonics).

### 2.7 Histological analysis

Kidney and cardiac tissues were fixed in 4% formaldehyde and embedded in paraffin. Tissue sections (4 μm) were stained using Periodic acid-Schiff (PAS) or Sirius Red and analyzed by a panel of board-certified pathologists.

#### 2.7.1 Kidney histopathology

Nephropathy severity was scored using Cohen Tervaerts’ classification [12] modified for mice (Supplemental Figure S3). Fifty contiguous glomeruli from a randomly selected region were analyzed and classified as normal, exhibiting mesangial matrix expansion, nodular sclerosis, segmental sclerosis (<75% of glomerular area) or global sclerosis (>75%). Interstitial fibrosis & tubular atrophy (IFTA) and inflammation were scored as described previously [12] on a scale of 0 (none), 1 (moderate) and 2 (advanced). For glomerular area measurements, PAS-stained sections were analyzed using Visiomorph software (Visiopharm, Hørsholm, Denmark).

For analysis of glomerular basement membrane (GBM) thickening, 4–15 transmission electron microscopy images were captured per mouse in healthy contralateral kidneys (t = −4 weeks) and from UNx + chow, UNx + HFD, UNx + HFD + L-NNA mice at t = 16 weeks and UNx + HFD + L-NNA mice at t = 20 weeks (study 2). GBM thickness was measured using ImageJ (version 1.53a; NIH, Bethesda, MD, USA). GBM width was measured orthogonally between the basal surfaces of the endothelium and podocyte foot processes.

#### 2.7.2 Cardiac histopathology

Cardiac fibrosis was scored blind on a 0–5 scale in the right and left ventricles, septum and perivascular areas by two observers. Average scores are reported.

### 2.8 Transcriptomics analysis

Kidney RNA was isolated from n = 9 UNx + chow mice and n = 7 kidneys in both HFD-fed groups. Quality control indicated overall good data quality. One UNx + HFD sample was excluded based on QC criteria. RNA-seq libraries were prepared using standard protocols and sequenced to generate 109–185 million paired-end, unstranded reads per sample (300 bp). No trimming was performed prior to alignment. Read quality and off-target content were assessed with FastQC and FastqScreen [13] (randomly sampled reads mapped to 11 organisms/artifacts. Reads were aligned to the Mus_musculus.GRCm38.91.gtf reference genome using STAR (version 2.5.3a), and distribution across annotated regions was inspected using picardtools.

Transcript annotation/quantification was performed in parallel using HTSeq [14] (version 1.99.2; gene level), Stringtie [15] (version 2.2.1; gene- and isoform level) and Kallisto [16] (version 0.46.1; isoform level). Expression data were normalized and analyzed on the log2 scale. PCA and Hierarchical clustering were used to identify outliers, which were removed when irregularities were consistent across measures. Voom [17]-transformed logCPM values from HTseq were used for downstream analyses. Pathway and upstream regulator analyses were performed using Ingenuity Pathway Analysis (IPA, Ingenuity Systems Inc., Redwood City, CA, USA).

### 2.9 Metabolome analysis

Plasma samples were mixed 1:5 with −20 °C LC-MS Grade methanol (047192-K2, ThermoFisher Scientific, Waltham, MA, USA) in low-protein-binding microcentrifuge tubes (#90410, ThermoFisher) and incubated overnight at −20 °C. After vortexing and centrifugation, supernatant was collected and a pooled QC sample was prepared. Extracts were dried, reconstituted in 0.2% formic acid, vortexed, sonicated and stored at −20 °C. Deproprionated plasma (7–10 μL) was analyzed by LC-MS/MS using Vanquish Horizon LC system coupled to a Q Exactive Plus Orbitrap MS (ThermoFisher) equipped with an Accucore Biphenyl column (17826–102130, ThermoFisher), as previously described [18]. Samples were run in both positive and negative ionization modes [18]. Feature identification and metabolite annotation were performed in Compound Discover (v3.3, ThermoFisher) with MS/MS-based matching against the mzCloud database. Metabolite intensities were normalized to levels at week 0.

### 2.10 Statistical analysis

Data were analyzed using SPSS (version 29.0.2.0, ICM Corp., Armonk, NY, USA). Data are presented as mean ± SEM. Group differences were determined by one-way ANOVA followed, when appropriate, by Tukey's multiple comparisons test. Two-tailed p-values are reported and p < 0.05 was considered statistically significant.

In Study 1, one UNx + HFD mouse reached humane endpoint (HEP) criteria at week 7. In the UNx + HFD + L-NNA group, one mouse reached HEP at week 4 and another died during anesthesia at week 15. No animals were lost in Study 2. In Study 3, one UNx + HFD mouse (week 5) and one UNx + HFD + L-NNA mouse (week 11) were found dead in the cage without prior signs of discomfort. Macroscopic evaluation revealed no abnormalities.

For transcriptomics, differentially expressed pathways (DEPs) and upstream regulators (URs) were selected based on pathway enrichment (p-value < 0.01, Fisher's exact test) and directionality (z-score). A z-score < −2 indicates predicted deactivation and z-score > 2 indicates predicted activation based on the direction of gene expression changes of target genes.

## 3 Results

### 3.1 Metabolic characterization of CKM mouse model: obesity, T2D and mild hyperlipidemia

**Figure 1:**
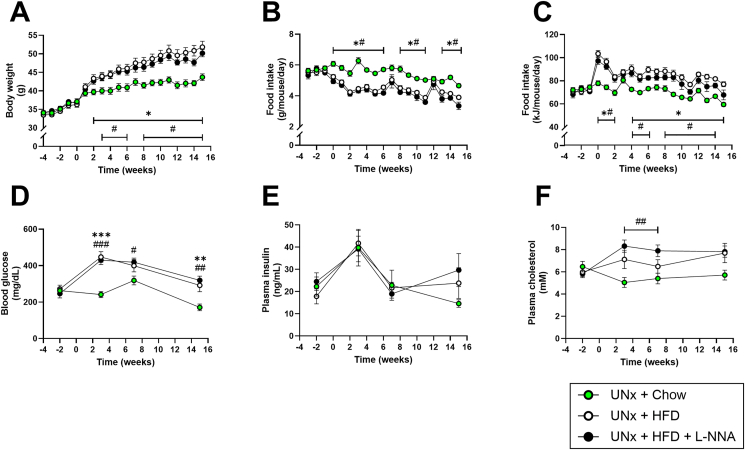
**High fat diet further increases body weight and blood glucose in uninephrectomized KK-Ay mice.** Body weight (A), daily food intake (g) (B), food intake (kJ) (C), blood glucose (D), plasma insulin (E) and plasma cholesterol (F). Data are presented as mean ± SEM for n = 9 in the UNx + chow group, n = 8 in the UNx + HFD group and n = 7 in the UNx + HFD + L-NNA group. ∗p < 0.05, ∗∗p < 0.01, ∗∗∗p < 0.001 UNx + HFD versus UNx + chow and ^#^p < 0.05, ^##^p < 0.01, ^###^p < 0.001 UNx + HFD + L-NNA versus UNx + chow. HFD: high fat diet; L-NNA: N^G^-Nitro-l-arginine hydrochloride; UNx: uninephrectomy.

Fifteen weeks of HFD feeding increased body weight compared to chow-fed mice (Figure 1A). Although food intake (g/day) was highest in UNx + chow mice (Figure 1B), energy intake (kCal/day) was higher in UNx + HFD and UNx + HFD + L-NNA groups (Figure 1C). At week −2, fasted blood glucose levels were already elevated (261 mg/dL [n.b.: >126 mg/dL is considered diabetic]) confirming spontaneous hyperglycemia. Glucose levels continued to rise to 439 mg/dL by week 3 in HFD-fed mice before gradually declining toward the study endpoint (Figure 1D). Plasma insulin was not affected by HFD (Figure 1E), whereas plasma cholesterol was significantly higher in UNx + HFD + L-NNA mice at several timepoints versus UNx + chow (Figure 1F).

In Study 3, body weight, food intake and glucose levels were comparable to Study 1 (Supplemental Figure S2A–C), although glucose in the UNx + HFD + L-NNA group was slightly lower than in UNx + HFD mice. Plasma insulin and cholesterol levels were higher overall in Study 3 (Supplemental Figure S2D + E). Together, these data demonstrate UNx, HFD and L-NNA synergistically amplify metabolic stress in KK-Ay mice, establishing the metabolic foundation for CKM progression.

### 3.2 CKM KK-Ay mice develop progressive renal failure characterized by early albuminuria, hyperfiltration and subsequent GFR decline

**Figure 2:**
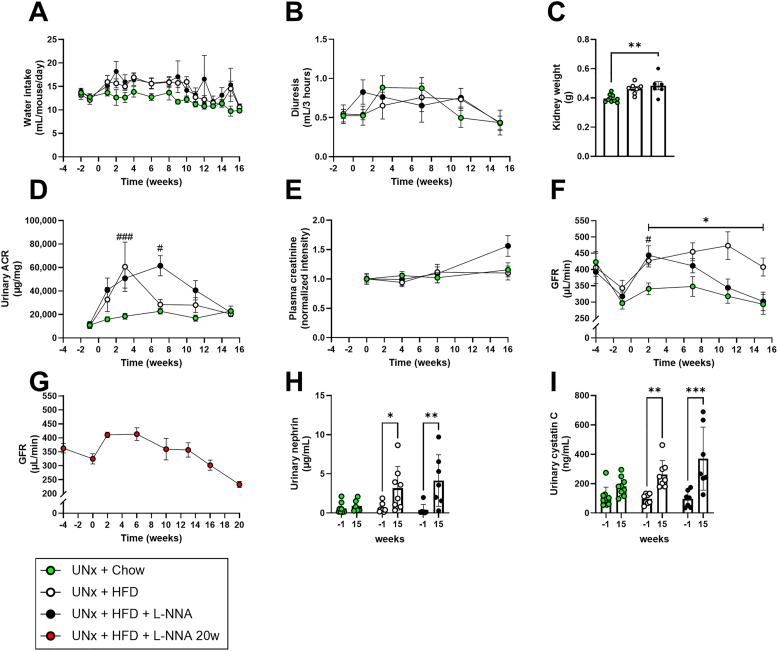
**Uninephrectomized KK-Ay mice on high fat diet and L-NNA develop progressive renal failure with a continuous decline in GFR.** Daily water intake (A), diuresis (B), kidney weight (C), urinary albumin/creatinine ratio (ACR) (D), plasma creatinine (E), glomerular filtration rate (GFR) (study 1) (F), GFR until 20 weeks (study 2) (G), urinary nephrin (H), urinary cystatin C (I). Data are presented as mean ± SEM for n = 9 in the UNx + chow group, n = 8 in the UNx + HFD group, n = 7 in the UNx + HFD + L-NNA group and n = 10 in the UNx + HFD + L-NNA 20 weeks group. ∗p < 0.05 UNx + HFD versus UNx + chow and ^#^p < 0.05, ^###^p < 0.001 UNx + HFD + L-NNA versus UNx + chow. HFD: high fat diet; L-NNA: N^G^-Nitro-l-arginine hydrochloride; UNx: uninephrectomy.

Given the metabolic disturbances and vulnerability of KK-Ay mice to renal complications, we next assessed whether these systemic changes translated into progressive renal dysfunction. As previously reported [19], water intake was elevated in all groups (Figure 2A), and diuresis remained comparable during the 3-h urine collection (Figure 2B). Kidney weight was significantly elevated in UNx + HFD + L-NNA mice (Figure 2C).

Urinary albumin creatinine ratio (UACR) was already elevated prior to dietary intervention, consistent with the KK-Ay background [20], and increased immediately following HFD initiation (Figure 2D). In UNx + HFD mice, UACR peaked at week 4 before declining, whereas in UNx + HFD + L-NNA mice UACR remained elevated until week 8 followed by a gradual decline. Plasma creatinine levels remained stable over time (Figure 2E), although UNx + HFD + L-NNA mice showed a nonsignificant upward trend at the endpoint (p = 0.11).

GFR, the gold-standard kidney function marker was subsequently assessed. In Study 1, baseline two-kidney GFR (week −5) and single-kidney GFR (week −1; three weeks post-UNx) were comparable across groups (Figure 2F). UNx + chow mice showed a transient rise in GFR that returned to baseline by week 15. Both HFD-fed groups exhibited immediate hyperfiltration. However, while UNx + HFD mice maintained elevated GFR, UNx + HFD + L-NNA mice showed a shorter hyperfiltration phase, followed by a progressive GFR decline from week 7 onward. Study 3 confirmed lower GFR at week 10 in UNx + HFD + L-NNA mice compared with UNx + HFD mice. In Study 2, UNx + HFD + L-NNA mice monitored for 20 weeks showed continued GFR decline, reaching a 44% reduction from peak hyperfiltration (Figure 2G). GFR values normalized to body weight for both Study 1 and Study 2 are shown in Supplemental Figure S3A + B, respectively.

Markers of renal injury supported these functional changes. Urinary nephrin [21], [22] was significantly elevated in both HFD-fed groups at week 15 (Figure 2H), and urinary cystatin C [23], [24] was similarly increased (Figure 2I). Metabolites linked to kidney disease progression also shifted, with p-cresol rising sharply in both HFD-fed groups, indicating reduced tubular clearance [25], 1-methylhistidine increasing most prominently in UNx + HFD + L-NNA mice, consistent with impaired amino acid handling [26], and kynurenic acid showing an early decrease followed by partial recovery, suggesting altered kynurenine-pathway activity [27] (Supplemental Figure S4A-C). Together, these findings demonstrate that UNx, HFD, and L-NNA induce early albuminuria, sustained hyperfiltration, and progressive renal failure accompanied by glomerular and tubular injury and metabolic signatures of declining renal clearance.

### 3.3 The CKM KK-Ay mouse model displays histological features of DKD, including glomerulosclerosis and tubulo-interstitial fibrosis

**Figure 3:**
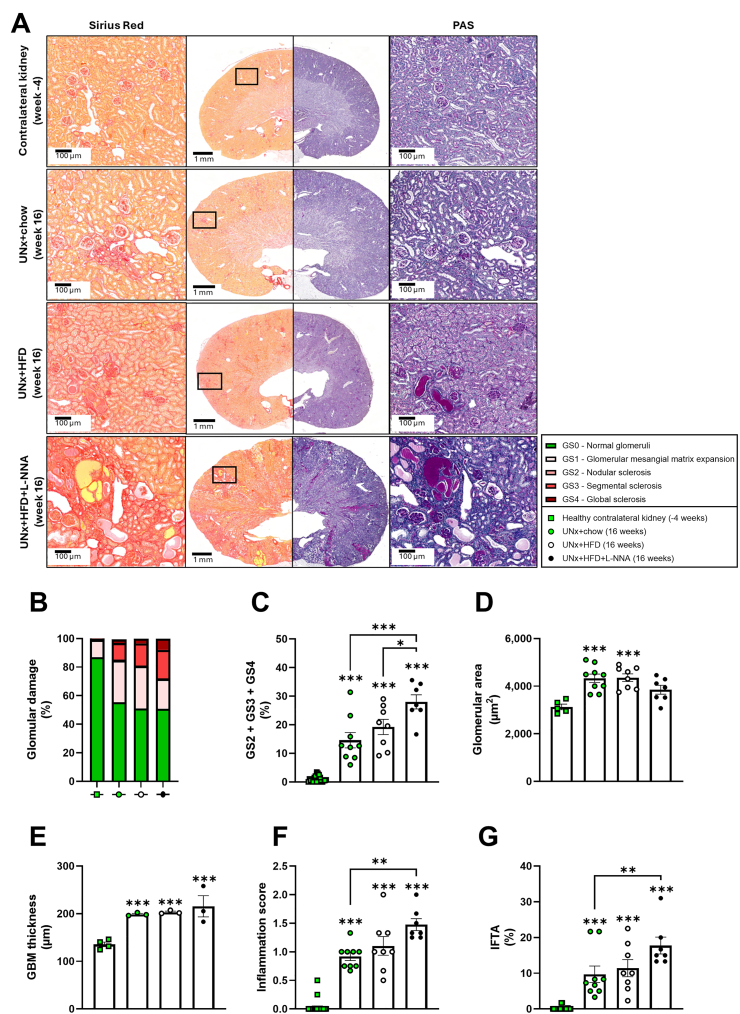
**Uninephrectomized KK-Ay mice on high fat diet and L-NNA display histological features of diabetic kidney disease, including glomerulosclerosis and tubulo-interstitial fibrosis.** Representative images of kidney sections stained with Sirius Red (left) and Periodic acid-Schiff (PAS) (right) (A). Renal histopathological scoring of these sections with fraction of damaged glomeruli (B), cumulative glomerular scoring (C), glomerular surface area (D), glomerular basement membrane (GBM) thickness (E), inflammation score (F) and interstitial fibrosis and tubular atrophy score (IFTA) (G). Data are presented as mean ± SEM for n = 24 contralateral kidneys removed during uninephrectomy at week −4, n = 9 in the UNx + chow group, n = 8 in the UNx + HFD group and n = 7 in the UNx + HFD + L-NNA group. ∗p < 0.05, ∗∗p < 0.01, ∗∗∗p < 0.001 UNx + HFD versus UNx + chow. HFD: high fat diet; L-NNA: N^G^-Nitro-l-arginine hydrochloride; UNx: uninephrectomy.

To determine whether functional and metabolic disturbances were accompanied by structural injury, renal histopathology was assessed. The mouse-adapted Cohen Tervaert scoring-system [12] (Supplemental Figure S5) was applied to healthy contralateral kidneys collected during UNx and to kidneys removed at the study endpoint (Figure 3A). Contralateral kidneys showed mostly normal glomeruli (Figure 3A+ B), whereas UNx + chow mice already exhibited mesangial expansion and some segmental and global sclerosis by week 16 (Figure 3A–C). Glomerular injury was greater in UNx + HFD mice and most pronounced in UNx + HFD + L-NNA mice, where nodular mesangial expansion and both segmental and global sclerosis were significantly increased compared with UNx + chow (+92%) and UNx + HFD (+46%) (Figure 3C). Automated image analysis revealed enlarged glomerular area in UNx + chow and UNx + HFD mice relative to contralateral kidneys, an effect absent in the UNx + HFD + L-NNA group (Figure 3D).

Additional DKD features, including Bowman's capsule dilation, arteriolar hyalinosis (Supplemental Figure S5), and GBM thickening (Figure 3E and Supplemental Figure S6) were present as well. Renal inflammation was abundant in UNx + chow and UNX + HFD mice and most pronounced in UNX + HFD + L-NNA mice (Figure 3F). Finally, interstitial fibrosis and tubular atrophy (IFTA) were absent in contralateral kidneys but were increased in UNx + chow and UNx + HFD mice and further aggravated by L-NNA mice (Figure 3G). Together, these findings show that UNx, HFD, and L-NNA drive progressive structural kidney damage characteristic of DKD.

### 3.4 Transcriptomic profiling reveals amplified inflammatory and fibrotic responses in UNx + HFD + L-NNA mouse kidneys

**Figure 4:**
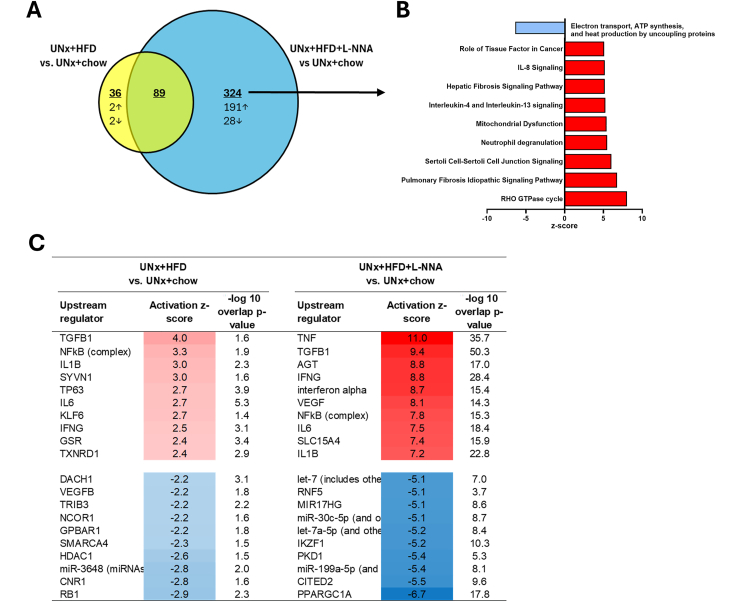
**Uninephrectomized mice on HFD and L-NNA activate pathways and upstream regulators involved in inflammatory processes.** Venn diagrams illustrate the overlap of differentially expressed pathways (DEPs) in kidney tissue between uninephrectomized (UNx) mice on high fat diet (HFD; n = 7, yellow) or UNx + HFD + L-NNA (n = 7, blue) compared to UNx + chow mice (n = 10) (A). The unique top 10 DEPs ranked by z-score and -log(p-value) are shown for the UNX + HFD + L-NNA group with blue bar indicating reduced activation and red bars indicating increased activation (B). Upstream regulator (UR) analysis illustrating the predicted activation state (z-score) of URs, based on target gene expression changes. Overlap p-values are reported and indicate significant overlap between the known target genes of a UR. The top 10 most significantly upregulated (red color) and top 10 most significantly downregulated (blue color) are shown for UNx + HFD and UNx + HFD + L-NNA versus UNx + chow (C).

To understand the mechanisms underlying the pronounced structural injury, we performed transcriptomic profiling of kidney tissue. Compared with UNx + chow mice, HFD feeding altered 125 pathways (Figure 4A; yellow part of the Venn diagram), 36 of which were unique, with only four showing strong activation or inhibition and the remaining pathways not showing clear directionality (−2.0 < z-score < 2.0). In contrast, compared to UNx + chow, UNx + HFD + L-NNA altered 413 pathways (blue part of the Venn diagram), including 324 unique DEPs, with 191 pathways upregulated (z-score > 2.0), 28 DEPs downregulated (z-score < −2) and 105 DEPs without clear directionality (−2.0<z-score<2.0). Eighty-nine DEPs overlapped between UNx + HFD and UNx + HFD + L-NNA, primarily involved in inflammation, cytoskeletal remodeling, fibrosis, and oxidative stress. Although significant in both groups, these pathways showed stronger enrichment and higher activation scores in UNx + HFD + L-NNA, suggesting that HFD in the UNx context primes the kidney, and L-NNA further amplifies inflammatory and metabolic dysregulation. Consistently, the top 10 DEPs in UNX + HFD + L-NNA versus UNX + chow highlight strong induction of inflammatory and fibrotic signaling, alongside suppressed mitochondrial metabolic activity (Figure 4B).

Upstream regulator (UR) analysis further supported this pattern (Figure 4C). In UNx + HFD mice, activated URs included pro-inflammatory mediators (TGFB1, NFkB, IL1B) and stress-related factors (TP63, GSR), while inhibited URs were primarily metabolic and chromatin-regulatory regulators (NCOR1, TRIB3, RB1). In the UNx + HFD + L-NNA group, UR activation was markedly stronger. The most strongly activated URs were several inflammatory mediators and VEGF, reflecting a compensatory angiogenic response to L-NNA-induced vasoconstriction. In UNx + HFD + L-NNA mice, inhibited URs included PPARGC1A, indicating reduced mitochondrial biogenesis, along with decreased activity of transcriptional regulators such as CITED2 and IKZF1.

### 3.5 Characterization of cardiac function in the CKM KK-Ay mouse model shows increased fractional shortening and ejection fraction

**Figure 5:**
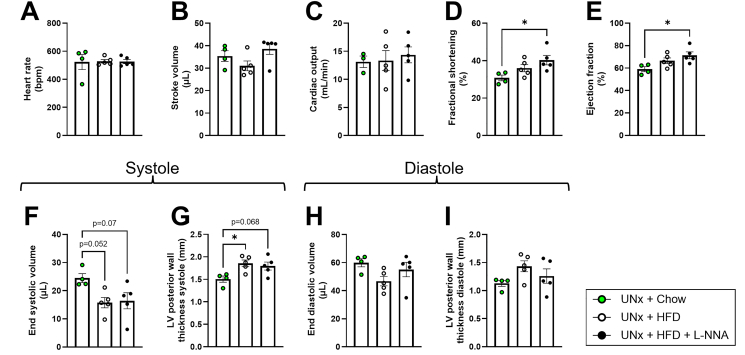
**Functional cardiac measurements point toward a phenotype resembling heart failure with preserved ejection fraction (HFpEF) in UNx + HFD + L-NNA mice.** Heart rate (A), stroke volume (B), cardiac output (C), fractional shortening (D), ejection fraction (E), end systolic volume during systole (F), left ventricular (LV) posterior wall thickness during systole (G), end diastolic volume (H) and LV posterior wall thickness during diastole (I). All data are presented as mean ± SEM for n = 5 mice per group. ∗p < 0.05. HFD: high fat diet; L-NNA: N^G^-Nitro-l-arginine hydrochloride.

Since metabolic and renal disturbances are intricately linked to cardiovascular remodeling in CKM syndrome, cardiac function was evaluated next. Heart rate, stroke volume, and cardiac output did not differ between groups (Figure 5A–C). In contrast, UNx + HFD + L-NNA mice showed increased fractional shortening and ejection fraction relative to chow controls (Figure 5D+ E). Both HFD-fed groups also showed a tendency toward reduced end-systolic volume (Figure 5F). Left ventricular (LV) posterior wall thickness during systole was significantly larger in UNx + HFD mice and showed a similar trend in UNx + HFD + L-NNA mice (Figure 5G). End-diastolic volume and LV posterior wall thickness during diastole were not different between groups (Figure 5H+I), nor were LV anterior wall thickness or LV inner diameter during systole or diastole (Supplemental Figure 7A–D). Collectively, these findings indicate that UNx + HFD + L-NNA KK-Ay mice exhibit enhanced LV contractility, reflected by increased fractional shortening and ejection fraction, accompanied by structural changes such as increased posterior wall thickness. This pattern is consistent with a compensatory hypertrophic response rather than improved cardiac function, aligning with early features of hypertrophic cardiomyopathy.

### 3.6 CKM KK-Ay mice develop histological features of cardiac fibrosis

**Figure 6:**
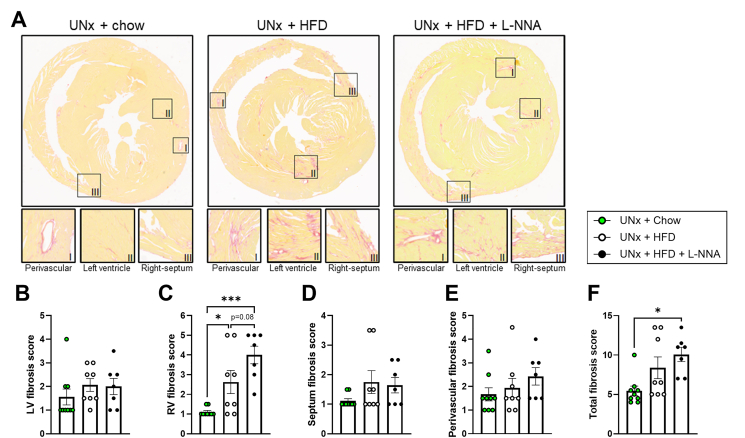
**Uninephrectomized KK-Ay mice on high fat diet and L-NNA develop cardiac fibrosis.** Sirius Red staining of heart cross-sections (A), left ventricular (LV) fibrosis score (B), right ventricular (RV) fibrosis score (C), septum fibrosis score (D), perivascular fibrosis score (E) and total fibrosis score (F). All data are presented as mean ± SEM for n = 9 in the UNx + chow group, n = 8 in the UNx + HFD group and n = 7 in the UNx + HFD + L-NNA group. ∗p < 0.05, ∗∗∗p < 0.001. HFD: high fat diet; L-NNA: N^G^-Nitro-l-arginine hydrochloride.

Given the observed functional alterations, we next investigated whether structural remodeling was also present. Cardiac fibrosis, a hallmark feature of heart failure and diabetic cardiomyopathy, was evaluated across groups (Figure 6A). LV fibrosis did not differ between groups (Figure 6B). In contrast, RV fibrosis was significantly increased in UNx + HFD mice (2.4-fold increase vs. UNx + chow) and further elevated in UNx + HFD + L-NNA mice (3.6-fold increase vs. UNx + chow), representing an additional 1.5-fold increase relative to UNx + HFD mice (Figure 6C). Septal and perivascular fibrosis were comparable across groups (Figure 6D–E). Overall, total cardiac fibrosis scores were highest in UNx + HFD + L-NNA mice (Figure 6F). Combined with the functional findings, these results might suggest a pattern of right-sided fibrotic remodeling that could be consistent with an HFpEF-like phenotype.

## 4 Discussion

This study establishes a multifactorial KK-Ay UNx + HFD + L-NNA mouse model that reproduces key features of CKM syndrome including obesity, T2D, progressive DKD and an HFpEF-like cardiac phenotype. Early onset of hyperglycemia and obesity provided a strong metabolic foundation on which UNx, HFD feeding and L-NNA-induced hypertension accelerated disease progression. These interventions induced renal injury consistent with clinical DKD/CKD [6,7]. These metabolic and renal disturbances contributed to a phenotype that might suggest HFpEF with preserved systolic function alongside concentric remodeling and increased RV fibrosis. By integrating metabolic, renal, and cardiac disease progression, this model provides a comprehensive platform for studying CKM pathophysiology and testing interventions.

Uninephrectomized KK-Ay mice developed obesity and T2D, a phenotype further aggravated by HFD and L-NNA, resulting in a patient-like metabolic profile that provides the context needed to model one of the key challenges in CKM research, namely progressive renal decline. Albuminuria, a key diagnostic criterion for DKD/CKD [28], is already present in KK-Ay mice [20,29,30]. In uninephrectomized mice, UACR remained high on chow, reflecting the baseline renal vulnerability of the model. Exposure to HFD with or without L-NNA, caused a pronounced additional increase in UACR, reaching levels that are considered extremely elevated. Therefore, AMDCC criteria [3], which recommend a >10-fold UACR increase compared to age- and sex-matched controls, were readily met. Furthermore, progressive renal failure, typically involving early hyperfiltration followed by gradual GFR decline, has been difficult to model preclinically. In UNx + HFD + L-NNA mice, GFR peaked at week 2 and then continuously decreased, a pattern confirmed in a 20-week study. Considering hyperfiltration as maximal renal capacity, GFR declined by 32% at 16 weeks and 44% at 20 weeks, aligning with AMDCC recommendations and clinical CKD definitions [3, 31]. As renal function declined, kidney-handled metabolites showed time-dependent alterations consistent with impaired renal processing and increased systemic cardiometabolic strain [32]. Together, these findings demonstrate that uninephrectomized KK-Ay mice on HFD, particularly with L-NNA, replicate key clinical features of progressive CKD, including substantial GFR decline.

Consistent with the GFR decline, UNx + HFD + L-NNA mice showed extensive glomerular damage, GBM thickening, arteriolar hyalinosis, and tubulo-interstitial fibrosis, characteristic of advanced DKD [33] and DKD progression in T2D patients [34]. Transcriptomics analyses corroborated these findings, with strong enrichment of inflammatory, fibrotic, and oxidative stress pathways along with activated upstream regulators such as TGFB1, NFKB, and IL1B and suppression of PPER1GC1A, indicating amplified immune and metabolic dysregulation. Unlike db/db mice, which mainly show metabolic and lipid-regulatory changes [35], this model emphasizes immune and fibrotic processes central to advanced kidney disease. This aligns with human CKD, where transcriptomic and multi-omics studies consistently identify inflammation, cytokine signaling, and extracellular matrix remodeling as key drivers of CKD and correlates with declining GFR [[36], [37], [38]].

Cardiac involvement, particularly HFpEF, is a defining feature of CKM syndrome, yet many unifactorial mouse models fail to capture its complexity [39]. Support for a causal link between CKD and HFpEF also comes from large-animal models, including a 5/6 nephrectomy model in which CKD induced preserved systolic function alongside diastolic impairment and structural cardiac remodeling, underscoring the central role of cardiorenal interactions in HFpEF pathogenesis [40]. Multifactorial approaches more closely resemble human HFpEF [39], especially when diabetes, an important but often overrepresented contributor to cardiovascular mortality, is incorporated [41,42]. Diabetes increases HFpEF-related mortality by 30–50% [43,44], and glucose-lowering interventions reduce HF incidence [45,46]. In this study, UNx + HFD induced LV posterior wall thickening and RV fibrosis, changes further amplified by L-NNA. Functionally, UNx + HFD + L-NNA mice showed concentric LV remodeling with preserved systolic function, while pronounced RV fibrosis, particularly at the insertion points, highlighted right-sided structural involvement. Although this pattern aligns with features commonly observed in HFpEF [47,48], definitive classification would require pulmonary hemodynamic assessment, which was not performed in the current study. Together, these findings demonstrate that this model recapitulates cardiac features pointing toward an HFpEF-like phenotype within a CKM context.

Over the years, several mouse models have been established to study obesity, diabetes, DKD, CKD and CVD, each with distinct strengths and limitations. Classic metabolic models such as db/db and ob/ob mice show robust obesity and hyperglycemia but lack strong cardiovascular involvement without additional interventions [49], whereas renin-AAV db/db and eNOS-knockout models provide strong renal and vascular phenotypes but rely on genetic manipulation, limiting translational applicability. In contrast to ob/ob and db/db mice with disrupted leptin signaling [50], KK-Ay mice retain an intact leptin receptor but develop leptin resistance through ectopic agouti protein-mediated antagonism of MC4R [51], making it a multifactorial model that naturally integrates metabolic, renal, and cardiac dysfunction. In the UNx + HFD + L-NNA form, these mice uniquely combine metabolic impairment, progressive CKD, and HFpEF-like remodeling without genetic modification. However, the strain is fragile and more challenging to house and handle due to severe hyperglycemia, which predisposes mice to skin lesions and impaired wound healing. Increased fighting behavior and stress sensitivity necessitates adjustments such as individual housing and minimizing handling procedures. Because handling and experimental procedures needed to be kept to an absolute minimum due to the strain vulnerability, these mice could undergo tail-cuff blood pressure measurements, as they easily overheat. Nevertheless, the efficacy of L-NNA supplementation on top of UNx + HFD was clearly demonstrated at the renal level, both histologically and at the transcriptomics level. In summary, the KK-Ay UNx + HFD + L-NNA model provides a unique multifactorial platform for studying CKM syndrome, capturing the progressive interaction of metabolic, renal, and cardiac dysfunction, including hallmark features such as obesity, T2D, CKD and HFpEF-like remodeling. By closely mirroring clinical disease progression, it enables detailed mechanistic studies while offering a robust framework for evaluating new therapies and advancing targeted interventions for CKM syndrome.

## CRediT authorship contribution statement

**Arianne van Koppen:** Writing – review & editing, Writing – original draft, Visualization, Validation, Supervision, Methodology, Funding acquisition, Formal analysis, Conceptualization. **José A. Inia:** Writing – review & editing, Writing – original draft, Visualization, Formal analysis, Data curation. **Romer A. Gonzalez-Villalobos:** Writing – review & editing, Validation, Methodology, Conceptualization. **Anke M. Smits:** Writing – review & editing, Investigation. **Andrea R. Nawrocki:** Writing – review & editing, Validation. **Simon A. Hinke:** Writing – review & editing, Validation, Methodology, Conceptualization. **Joline Attema:** Writing – review & editing, Investigation. **Christa de Ruiter:** Writing – review & editing, Investigation. **Tri Q. Nguyen:** Writing – review & editing, Methodology, Conceptualization. **Amelie Dendooven:** Writing – review & editing, Methodology, Conceptualization. **Ingeborg Bajema:** Writing – review & editing, Methodology, Conceptualization. **Harry van Goor:** Writing – review & editing, Software, Methodology, Conceptualization. **Toon A.B. van Veen:** Writing – review & editing, Investigation. **Willem B. van Ham:** Writing – review & editing, Investigation. **Felix Eichinger:** Writing – review & editing, Validation. **Søren H. Elsborg:** Writing – review & editing, Validation. **Henricus A.M. Mutsaers:** Writing – review & editing, Validation. **Elsbet J. Pieterman:** Writing – review & editing, Conceptualization. **Aswin L. Menke:** Writing – review & editing, Investigation. **Matthew D. Breyer:** Writing – review & editing, Validation, Methodology, Conceptualization. **Reinout Stoop:** Writing – review & editing, Writing – original draft, Visualization, Supervision, Project administration, Investigation, Funding acquisition, Formal analysis, Conceptualization.

## Disclosures

R.A.G.V., A.R.N., S.A.H., and M.D.B. were employees and stockholders of Johnson & Johnson at the time the project was conducted. The other authors have no conflicts of interest to disclose.

## Funding

This collaboration project is co-funded by the PPP Allowance made available by Health-Holland, Top Sector Life Sciences & Health to stimulate public-private partnership grant nr V201700972.

## Declaration of competing interest

The authors declare the following financial interests/personal relationships which may be considered as potential competing interests: All authors reports financial support was provided by Health-Holland, Top Sector Life Sciences & Health. Romer A. Gonzalez-Villalobos reports a relationship with Johnson & Johnson that includes: employment and equity or stocks. Andrea R. Nawrocki reports a relationship with Johnson & Johnson that includes: employment and equity or stocks. Simon A. Hinke reports a relationship with Johnson & Johnson that includes: employment and equity or stocks. Matthew D. Breyer reports a relationship with Johnson & Johnson that includes: employment and equity or stocks. R.A.G.V., A.R.N., S.A.H., and M.D.B. were employees and stockholders of Johnson & Johnson at the time the project was conducted. The other authors have no conflicts of interest to disclose. If there are other authors, they declare that they have no known competing financial interests or personal relationships that could have appeared to influence the work reported in this paper.

## Data Availability

Data will be made available on request.
