# Supplementary material for: A novel mouse model for cardiovascular-kidney-metabolic syndrome: Bridging metabolic, renal and cardiac dysfunction
Source: Mol Metab. 2026 Apr 20;108:102368. doi: 10.1016/j.molmet.2026.102368 (PMC13145895; doi:10.1016/j.molmet.2026.102368)
Supplement: Multimedia component 1 [file mmc1.docx]

# Supplemental material

A novel mouse model for cardiovascular-kidney-metabolic syndrome: bridging metabolic, renal and cardiac dysfunction

Arianne van Koppen, José A. Inia, Romer A. Gonzalez-Villalobos, Anke M. Smits, Andrea R. Nawrocki, Simon A. Hinke, Joline Attema, Christa de Ruiter, Tri Q. Nguyen, Amelie Dendooven, Ingeborg Bajema, Harry van Goor, Toon A.B. van Veen, Willem B. van Ham, Felix Eichinger, Søren H. Elsborg, Henricus A.M. Mutsaers, Elsbet J. Pieterman, Aswin L. Menke, Matthew D. Breyer, Reinout Stoop

## Supplemental figures


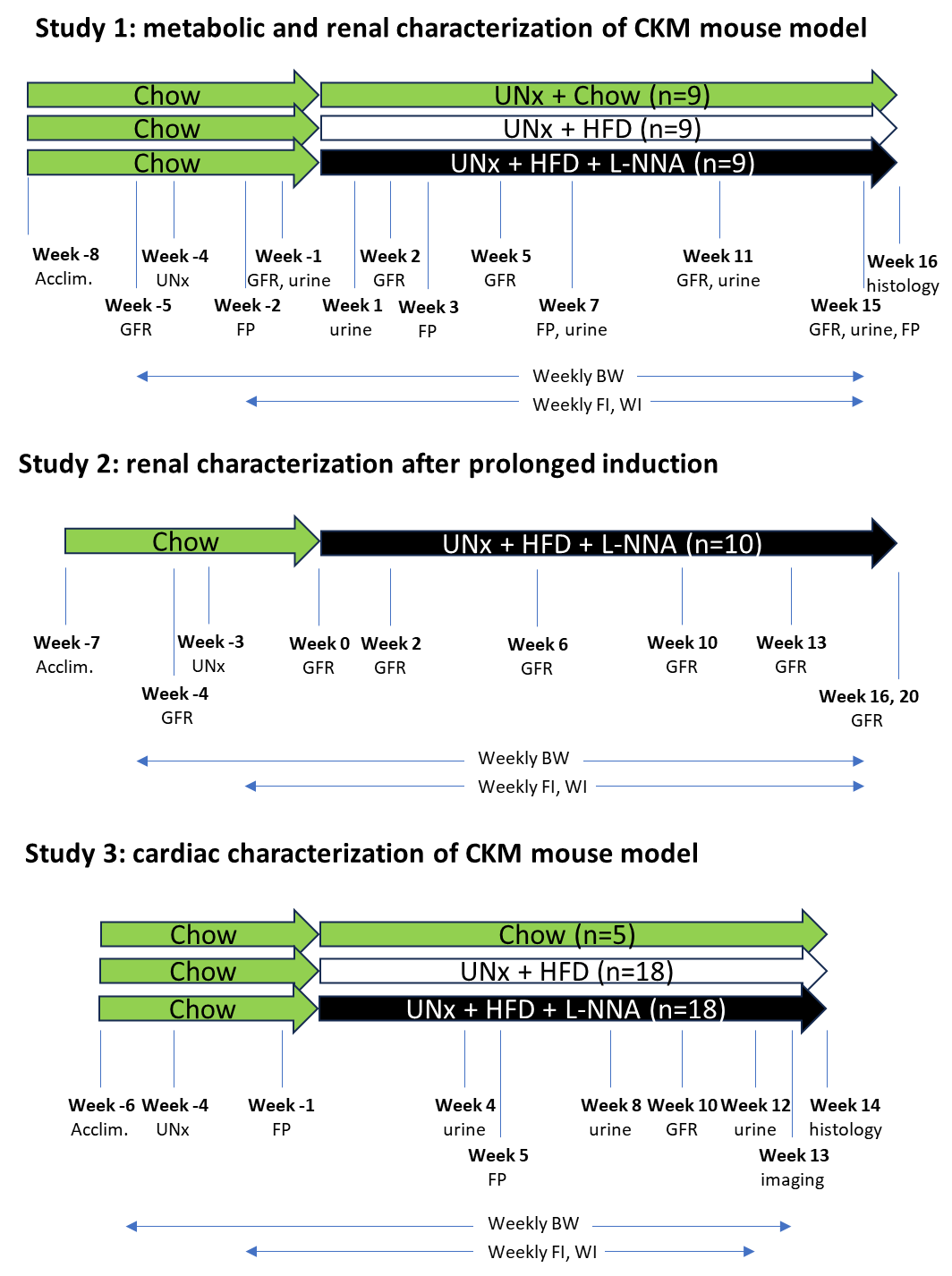


**Supplemental Fig. S1**. **Study outlines.** KK-Ay mice underwent uninephrectomy (UNx) at week -4/-3. At week 0, mice were matched into equal groups and continued on the healthy chow diet or were switched to high fat diet (HFD) with or without 50 mg L-NNA in the drinking water. Acclim: acclimatization; BW: body weight; CKM: cardiovascular-kidney-metabolic syndrome; FI: food intake; FP: fasted plasma; GFR: glomerular filtration rate; L-NNA: N^G^-Nitro-L-arginine hydrochloride; UNx: uninephrectomy; WI: water intake.


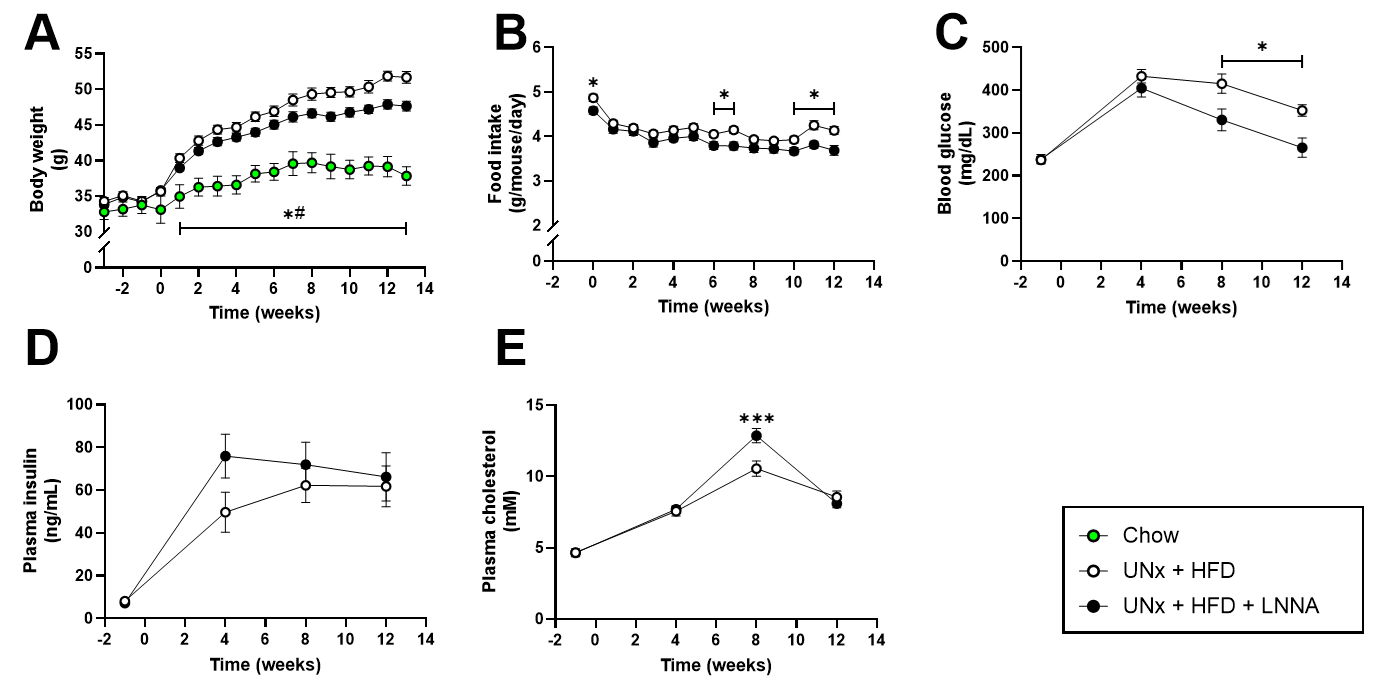


**Supplemental Fig. S2. Metabolic parameters in the cardiac characterization study (study 3).** Body weight (A), daily food intake (B), blood glucose (C), plasma insulin (D) and plasma cholesterol (E). Data are presented as mean ± SEM for n=4 mice in the chow-fed group and n=17 mice in the HFD-fed groups. *p<0.05, ***p<0.001 UNx+HFD versus UNx+chow and ^#^p<0.05 UNx+HFD+L-NNA versus UNx+chow. HFD: high fat diet; L-NNA: N^G^-Nitro-L-arginine hydrochloride; UNx: uninephrectomy.

**
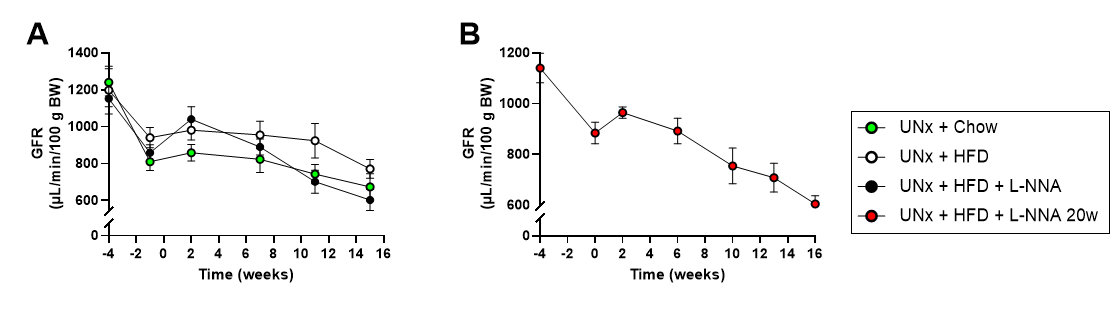
**

**Supplemental Fig. S3. Normalized GFR levels over time.** Glomerular filtration rate (GFR) normalized per 100 g body weight (study 1) (A) and GFR normalized per 100 g body weight (study 2) (B). Data are presented as mean ± SEM for n=9 mice per group. BW: body weight; HFD: high fat diet; L-NNA: N^G^-Nitro-L-arginine hydrochloride; UNx: uninephrectomy.

**
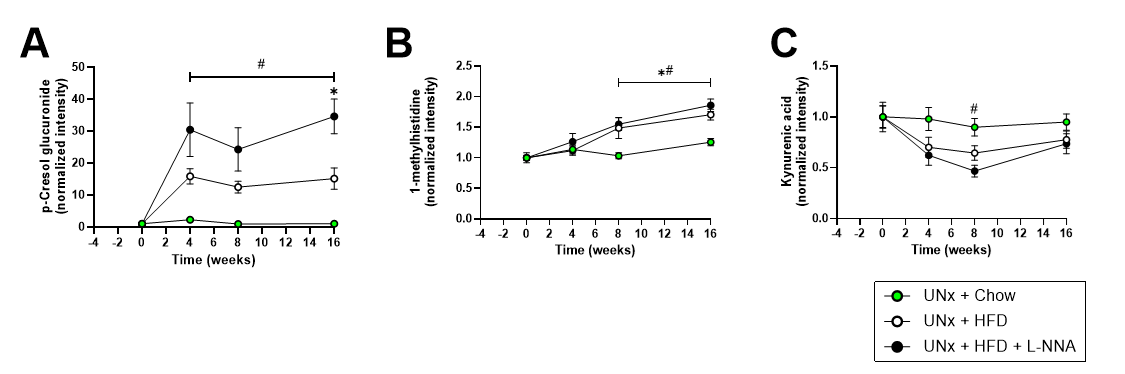
**

**Supplemental Fig. S4. HFD feeding and L-NNA alter plasma metabolites.** P-cresol glucuronide (A), 1-methylhistidine (B) and kynurenic acid (C). Data are presented as mean±SEM for n=9 mice per group. *p<0.05 UNx+HFD versus UNx+chow and ^#^p<0.05 UNx+HFD+L-NNA versus UNx+chow. HFD: high fat diet; L-NNA: N^G^-Nitro-L-arginine hydrochloride; UNx: uninephrectomy.


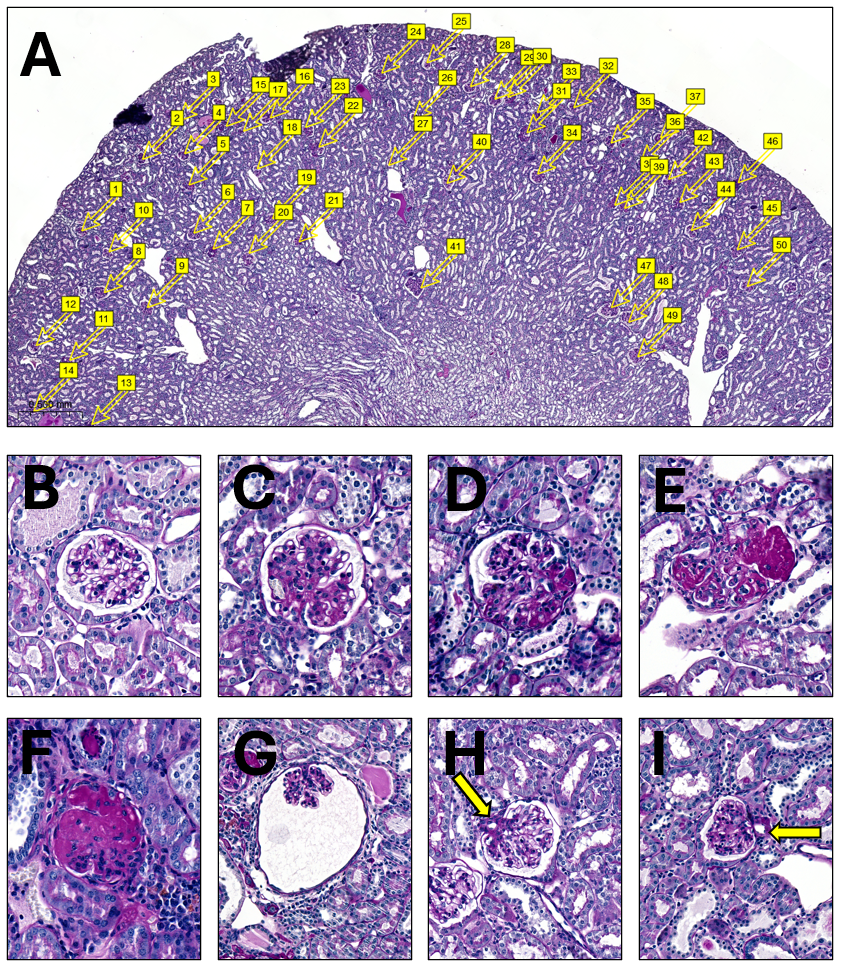


**Supplemental Fig. S5. Histopathological assessment of PAS-stained glomeruli.** Fifty contiguous glomeruli were annotated randomly. Subsequently, each glomerulus was histologically assessed whether it appeared normal (B) or showed mesangial matrix expansion (C), nodular sclerosis (D), segmental sclerosis (E), global sclerosis (F), a dilated Bowman’s capsule (G) or arteriolar hyalinosis (H, I arrows), using the mouse-adapted Cohen Tervaert classification score^1^.

1. Tervaert TWC, Mooyaart AL, Amann K, et al. Pathologic classification of diabetic nephropathy. *Journal of the American Society of Nephrology*. 2010;21(4):556-563.


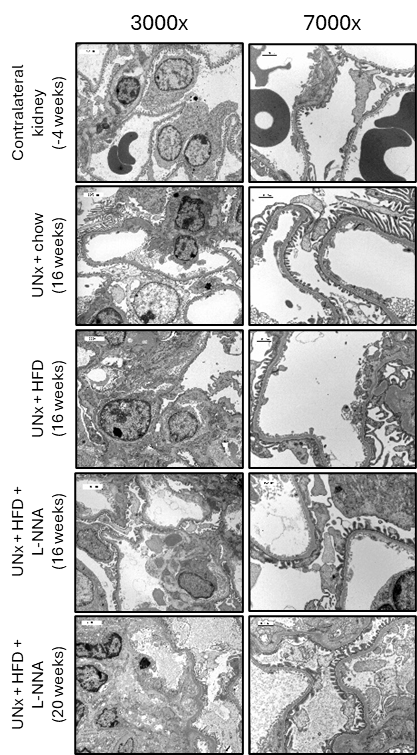


**Supplemental Fig. S6. Transmission electron microscopy images of the glomerular basement membrane.** TEM images were made at 3000x and 7000x magnification for healthy contralateral kidneys removed during uninephrectomy (t=-4 weeks) and for mice in the UNx+chow, UNx+HFD and UNx+HFD+L-NNA groups sacrificed at t=16 weeks and for mice in the UNx+HFD+L-NNA group sacrificed at t=20 weeks. HFD: high fat diet; L-NNA: N^G^-Nitro-L-arginine hydrochloride; UNx: uninephrectomy.


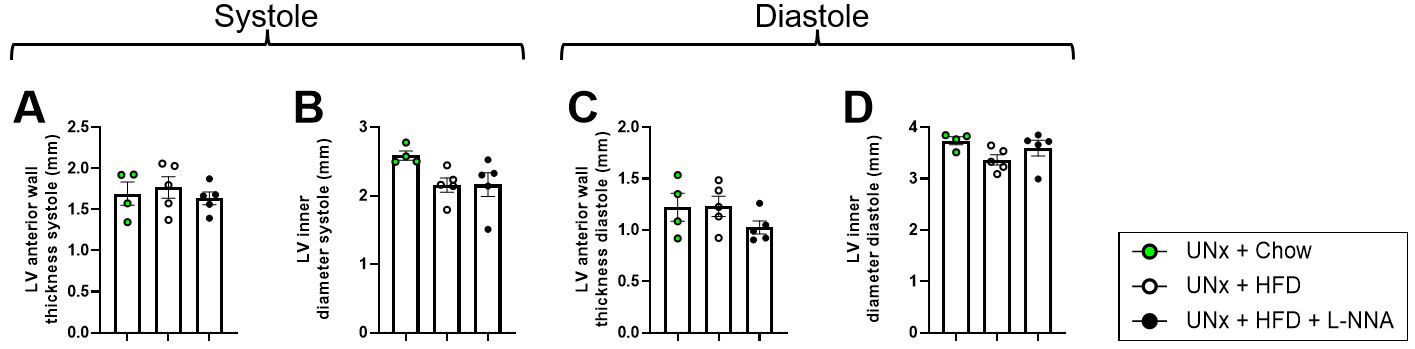


**Supplemental Fig. S7. Cardiac function in uninephrectomized KK-Ay mice.** Left ventricular (LV) anterior wall thickness during systole (A), LV inner diameter during systole (B), LV anterior wall thickness during diastole (C) and LV inner diameter during systole (D). Data are presented as mean ± SEM for n=4 mice in the chow-fed group and n=5 mice in the HFD-fed groups. HFD: high fat diet; L-NNA: N^G^-Nitro-L-arginine hydrochloride; UNx: uninephrectomy.
